# Dissecting neuronal circuit function and dysfunction in Alzheimer’s disease mouse models: from conventional calcium imaging analysis to machine learning-based approaches

**DOI:** 10.3389/fnagi.2026.1930851

**Published:** 2026-09-11

**Authors:** Evgenii Gerasimov, Evgenii Fedorov, Ilya Bezprozvanny, Ekaterina Pchitskaya

**Affiliations:** 1 Laboratory of Molecular Neurodegeneration, Peter the Great St. Petersburg Polytechnic University, Saint Petersburg, Russia; 2 Laboratory of Molecular Neurobiology, Pavlov Institute of Physiology, Russian Academy of Sciences, Saint Petersburg, Russia; 3 Laboratory of Biomedical Imaging and Data Analysis, Peter the Great St. Petersburg Polytechnic University, Saint Petersburg, Russia; 4 School of Basic Medical Sciences, Inner Mongolia Medical University, Hohhot, Inner Mongolia, China

**Keywords:** Alzheimer’s disease, artificial intelligence, *in vivo* calcium imaging, machine learning, miniscope, neuronal circuit dysfunction, two-photon calcium imaging

## Abstract

*In vivo* calcium imaging is a powerful technique for the monitoring activity of large neuronal populations in the intact brain. Two-photon microscopy provides subcellular resolution in head-fixed animals, and miniaturized fluorescence microscopy (miniscope) enables recordings of neuronal network activity in freely behaving animals. Combined with genetically encoded calcium indicators, these methods have revealed important new information about the functioning of neuronal circuits and enabled investigation of neuronal network dysfunction in mouse models of Alzheimer’s disease. Typically, such imaging data are analyzed using event-based and statistical approaches, which have been highly informative but fail to capture the higher-order spatiotemporal structure of circuit activity. Here we propose that dimensionality reduction and neuronal manifold analysis, machine learning, and artificial intelligence (AI)-based methods provide powerful data-driven approaches, enabling feature extraction, detection of disease-associated network states, and simultaneous analysis of neural network activity and behavior. Crucially, beyond describing pathology, these approaches could offer a more sensitive and complementary readout for screening candidate therapeutic strategies in neurological disease models. Here, we summarize neuronal activity alterations identified across Alzheimer’s disease mouse models using classical and AI-based calcium imaging analyses, and outline future perspectives for AI-driven approaches in this field, from interpretable architectures to multimodal integration of activity and behavior.

## 1 Introduction

Advanced technologies for neuronal activity recording have revolutionized the understanding of brain function under both physiological and pathological conditions (Russell, 2011; Werner et al., 2019; Grienberger et al., 2022; Guo et al., 2023). Concurrently, advances in optical imaging modalities, such as multiphoton, confocal (Svoboda and Yasuda, 2006; Grienberger et al., 2022; Zong et al., 2022; Xiong et al., 2023), and miniaturized fluorescence microscopy (Aharoni and Hoogland, 2019; Gerasimov et al., 2020), have enabled researchers to conduct *in vivo* calcium imaging experiments in freely behaving animals. Head-fixed two-photon calcium imaging provides high spatial resolution, optical sectioning, and reduced background fluorescence, enabling reliable recording of neuronal activity at cellular and subcellular resolution (Helmchen and Denk, 2005; Grienberger et al., 2022; Zong et al., 2022). However, this approach typically requires animals to remain head-fixed, restricting behavioral paradigms to constrained or virtual environments and limiting recordings primarily to optically accessible brain regions. In contrast, head-mounted single-photon miniscopes enable longitudinal imaging of neuronal populations in freely behaving animals (Kingsbury et al., 2019; Shuman et al., 2020; Barry et al., 2022; Gerasimov et al., 2024), including deep brain structures such as the hippocampus through implanted GRIN lenses, albeit with lower spatial resolution, greater out-of-focus fluorescence, and reduced optical sectioning (Barbera et al., 2022; Chen et al., 2022). More recently, head-mounted two-photon miniscopes have combined the advantages of two-photon excitation with imaging in freely moving animals, although their greater weight and complexity, as well as limited availability currently restrict their widespread use (Zhao et al., 2023; Madruga et al., 2025; Zhang et al., 2025). Given these advantages, single-photon miniscope imaging has become an effective tool for investigating the organization and function of neuronal circuits and networks, as well as their roles in memory (Cai et al., 2016; Zaki et al., 2025; de Sousa et al., 2026), navigation (Rubin et al., 2019), and other cognitive processes under behaviorally relevant conditions (Kingsbury et al., 2019; Hur et al., 2024). Furthermore, miniscope imaging has also proven to be a crucial method for studying neuronal and circuit dysfunction in Alzheimer’s disease, as it allows pathological processes to be captured at both the individual cell and population levels (Werner et al., 2019; Lin et al., 2022; Zhang et al., 2023; Gerasimov et al., 2025b).

In two photon and miniscope *in vivo* calcium imaging recordings the raw data output has the same form – a fluorescence recording that, after processing, yields per-neuron calcium traces and their spatial footprints (Zhou et al., 2018; Giovannucci et al., 2019; Cantu et al., 2020; Dong et al., 2022). However, these methods enable extraction of different types of information about neuronal activity, depends on the analytical algorithms employed. There has been rapid development of analysis protocols used to process this type of data, ranging from classical statistical methods to machine learning (ML) and artificial intelligence (AI)-based platforms that offer promising opportunities for automated feature extraction, detection of hidden activity patterns, prediction of disease-associated network states, and integration of multimodal datasets, potentially deepening our understanding of neuronal network function and dysfunction. Here, we review these approaches in the context of Alzheimer’s disease mouse models, first outlining the standard processing pipeline for calcium imaging data and the event-based and statistical methods built upon it, then surveying emerging computational strategies, including manifold learning, information-theoretic analysis, and AI models that extend these frameworks. Further, we discuss specific applications of these analysis approaches to studies of neuronal network dysfunction across several Alzheimer’s disease mouse models, highlighting both areas of consensus and sources of variability in the reported findings. In the final section we summarize the prospects for applying AI methods to *in vivo* calcium imaging data and highlight the challenges and unresolved questions associated with this approach.

## 2 General pipeline for the analysis of *in vivo* calcium imaging data

**FIGURE 1:**
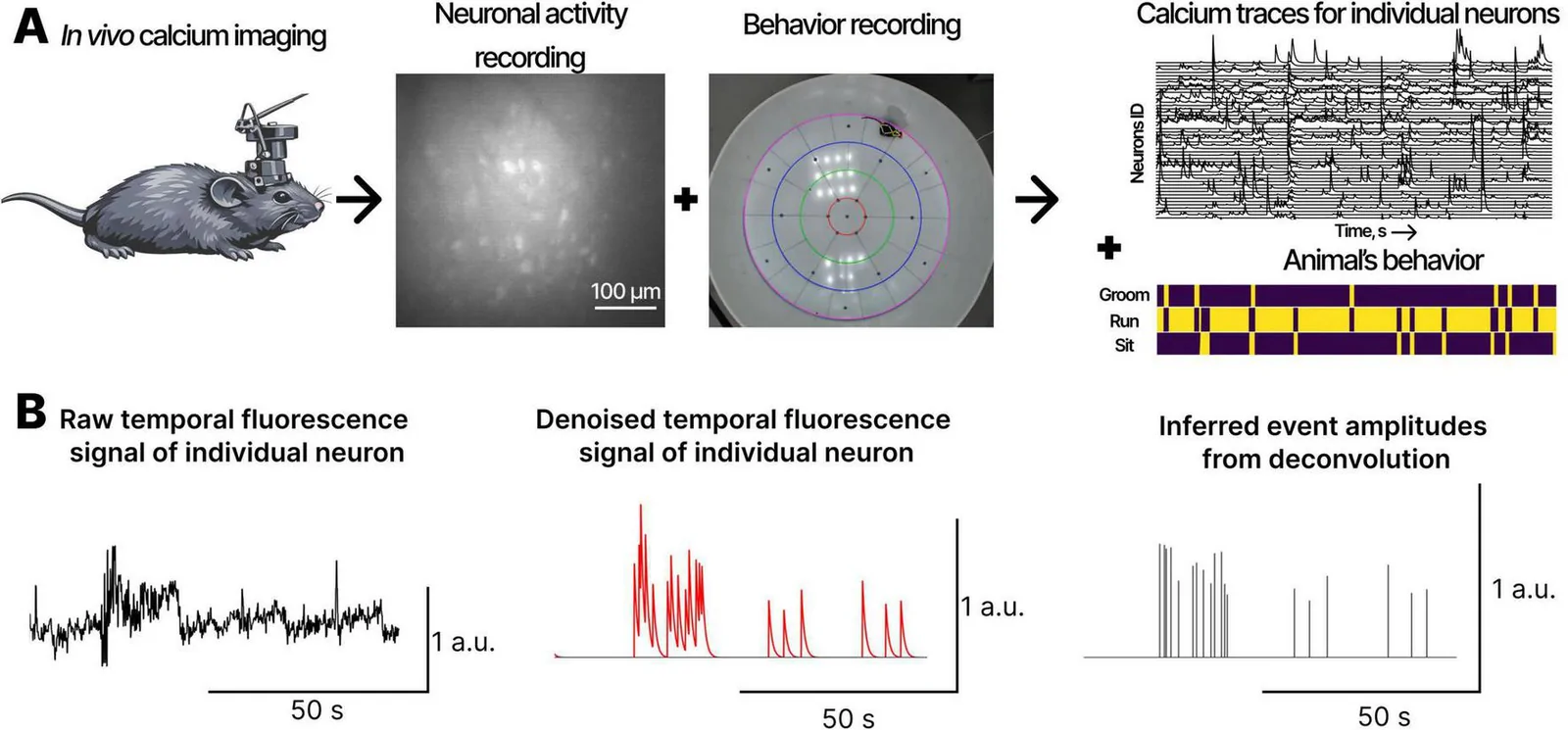
*In vivo* calcium imaging pipeline and signal representation. **(A)** Pipeline for *in vivo* recording of neuronal activity using miniature fluorescence microscopy-based calcium imaging. **(B)** Calcium imaging signal formats (left to right): raw temporal fluorescence, denoised temporal fluorescence, and inferred event amplitudes for individual neuron.

Miniscope imaging data are typically represented as video recordings of fluorescence signals from a targeted brain region, where changes in fluorescence intensity are driven by intracellular Ca^2 +^ concentration changes associated with neuronal excitation, synaptic input, and action-potential-related calcium influx (Pologruto et al., 2004; Chen et al., 2013; Figure 1A). Following initial processing of these recordings, spatial footprints are extracted, corresponding to regions of interest (ROIs) associated with individual neurons (Zhou et al., 2018; Giovannucci et al., 2019; Cantu et al., 2020; Dong et al., 2022). For each ROI, a temporal signal is then generated, referred to as a calcium trace, which reflects changes in neuronal fluorescence over time (Figure 1A). These data are typically represented in several forms, including raw fluorescence signals, ΔF/F traces reflecting relative changes from baseline fluorescence, and inferred spike events obtained through deconvolution of the calcium signal (Figure 1B; Deneux et al., 2016; Pachitariu et al., 2018). Different representations of calcium imaging data provide complementary perspectives on neuronal activity (Figure 1B). Raw fluorescence preserves the original imaging signal and is useful for assessing baseline intensity and signal quality, whereas ΔF/F normalization enables comparison across neurons by accounting for differences in indicator expression and imaging conditions, although it remains influenced by noise and calcium kinetics (Oh et al., 2019). Deconvolved spike estimates aim to infer underlying neuronal firing events from calcium dynamics, offering improved temporal interpretation of activity patterns but relying on model assumptions and limited by the slow and non-linear relationship between calcium signals and action potentials (Deneux et al., 2016; Pachitariu et al., 2018). However, it is important to emphasize that none of these calcium trace representations constitutes a direct recording of action potentials; rather, they reflect slower and smoother optical signals that correlate with neuronal activity.

For miniscope calcium imaging data, classical analysis pipelines typically begin with the extraction of reliable and reproducible cellular signals. This type of processing is implemented in widely used pipelines such as CNMF-E (Zhou et al., 2018), CaImAn (Giovannucci et al., 2019), MIN1PIPE (Lu et al., 2018), EZcalcium (Cantu et al., 2020), and Minian (Dong et al., 2022), where the principal steps include motion correction, source extraction, component quality assessment, and generation of finalized raw or deconvolved calcium signals. Finally, fluorescence intensity traces over time and spatial footprints coordinates are obtained for each extracted neuron, enabling further analysis.

At the single-cell level, activity analysis is usually interpreted using predefined metrics, including the frequency of calcium events, the amplitude and duration of calcium transients, the mean activity level, and the proportions of hyperactive and hypoactive cells. These metrics have been widely used to investigate both physiological brain function and neuropathological conditions, particularly in Alzheimer’s disease mouse models, where they are applied to characterize disruptions associated with neurodegenerative processes. At the population level, classical analytical methods generally involve the construction of activity raster plots, estimation of coactivity intervals, and calculation of interneuronal correlations. These methods enable detection of impairments in the coherence of neuronal population activity and pathological rearrangements of functional relationships between cells. Another important aspect of calcium imaging data analysis is the investigation of the relationship between cellular activity and locomotion and behavior, which commonly involves the construction of activity maps, calculation of spatial information, assessment of place-field stability, evaluation of response reliability across trials, and analysis of pathological changes in these characteristics. Application of these strategies to disrupted neuronal circuit function in Alzheimer’s disease will be demonstrated in section “4 Traditional analytical approaches applications for *in vivo* calcium imaging data analysis for assessing neural circuits activity dysfunction in Alzheimer’s disease mouse models.”

## 3 Emerging computational strategies for decoding neural activity from calcium imaging data

**FIGURE 2:**
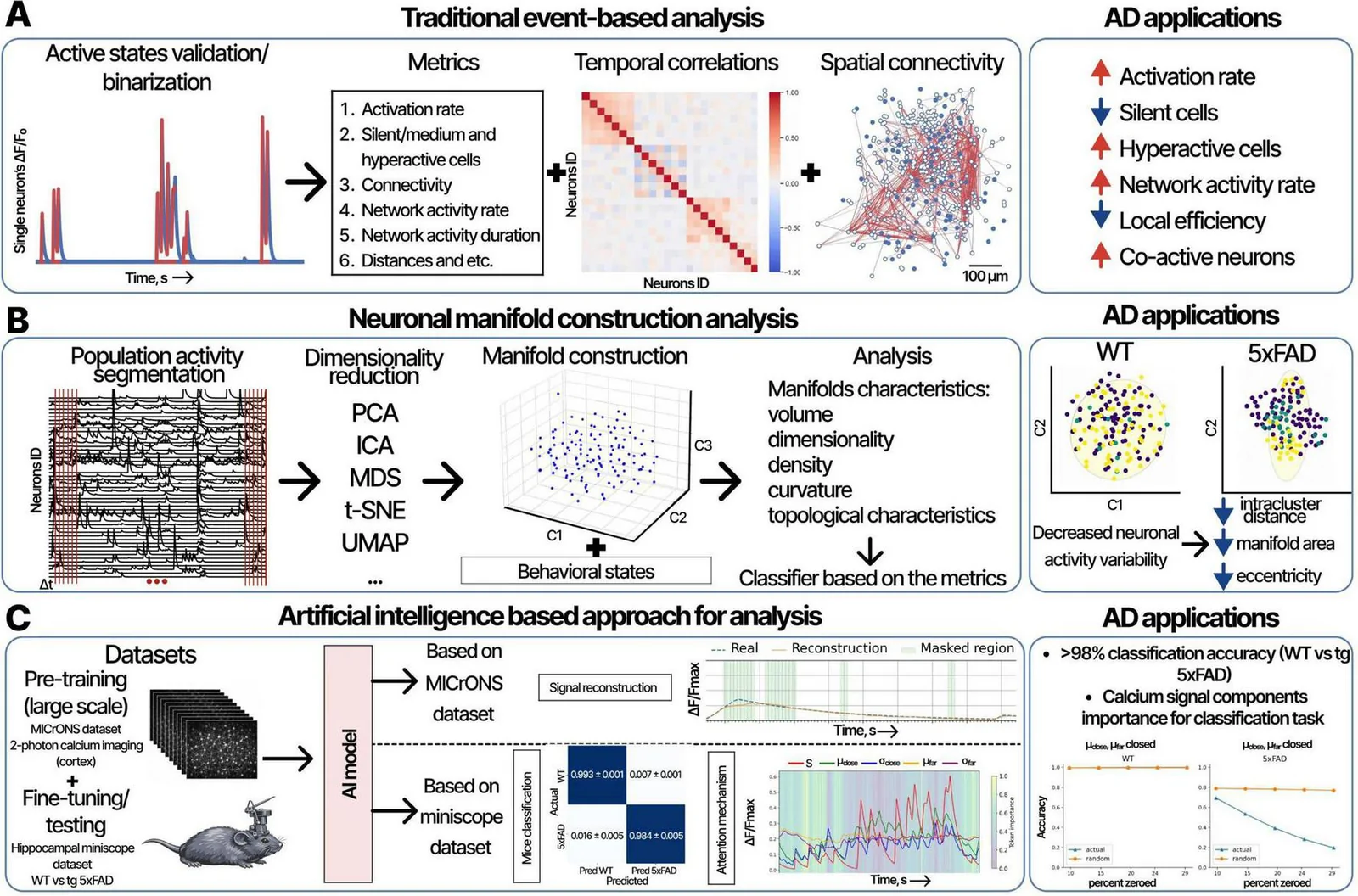
Methods of analysis of neuronal network activity as revealed by *in vivo* calcium imaging data and their application to analysis of data obtained with AD mouse models. **(A)** Workflow of traditional event-based analyses and their application to the study of neuronal circuit dysfunction. **(B)** Schematic representation of neuronal manifold construction and analysis for characterizing disruptions in population-level neuronal activity. Figures for “Manifold construction” and “AD application” in this section are partly adapted from Gerasimov et al. (2025a). **(C)** Framework illustrating the application of artificial intelligence methods to investigate neuronal network function in mouse models from calcium imaging datasets. Figures for “Signal reconstruction,” “Attention mechanisms,” and “AD applications” in the current section are partly adapted from Raev et al. (2026). Copyright the authors, Georgii Raev, Daniil Baev, Evgenii Gerasimov, Viacheslav Chukanov, Ekaterina Pchitskaya. AD, Alzheimer’s disease; AI, artificial intelligence; C1, C2, C3, first, second, and third component in reduced dimension; ΔF/F_0_, relative change in fluorescence intensity; ΔF/F_max_, fluorescence signal normalized to maximum fluorescence; ICA, independent component analysis; MDS, multidimensional scaling; PCA, principal component analysis; t-SNE, t-distributed stochastic neighbor embedding; UMAP, uniform manifold approximation and projection; WT, wild type; 5xFAD, five familial Alzheimer’s disease mutations transgenic mouse model.

Classical event-based analysis provides valuable characteristics of specific aspects of neuronal activity, such as activation rates and pairwise co-activation patterns (Figure 2A). A range of computational tools has been developed to transform processed calcium traces into interpretable measures of cellular and network activity. Mesmerize, for example, is an interactive environment that combines processing, visual inspection, annotation, and analysis of two- and three-dimensional calcium-imaging data (Kolar et al., 2021). It also allows neural signals to be linked to time-aligned experimental conditions, stimuli, and behavioral variables (Klumpp et al., 2025). NeuroActivityToolkit, developed by our laboratory, provides a complementary set of miniscope calcium-imaging metrics, including neuronal activations count, co-active cells number, and pairwise correlations (Gerasimov et al., 2023). Such approaches are particularly useful for comparing cellular and network activity under normal and pathological conditions, including models of neurodegeneration.

However, despite producing valuable results, traditional analytical approaches are often limited in their ability to capture the collective dynamics of neuronal populations as high-dimensional systems. First, calcium signals are an indirect and temporally smoothed proxy of neuronal activity, meaning that the underlying patterns of neural firing must be inferred rather than directly observed. Second, neuronal populations exhibit latent organization in the form of coordinated, low-dimensional dynamics that cannot be fully described by pairwise activity measures or a limited set of predefined parameters. Third, in calcium imaging experiments performed in freely moving animals, population activity is strongly shaped by behavior, whereas predefined metrics generally treat behavior only as an external covariate. Although classical analytical approaches have significantly improved our understanding of neuronal network function under normal and pathological conditions, they typically decompose neural activity into separate quantitative features. As a result, they may represent only limited aspects of complex neural states, multicellular coordination patterns, information encoding, and the relationship between neuronal dynamics and behavior. Consequently, emerging analytical frameworks increasingly address these limitations by treating neuronal population activity as a complex, high-dimensional dynamical system.

The computational approaches described below partly address these gaps and can be divided into four broad methodological families according to their principal analytical objectives. The first includes methods that reconstruct latent physiological processes or functional interactions from fluorescence signals, including neural firing, calcium dynamics, and functional-network organization (Lütcke et al., 2013; Rahmati et al., 2016). The second comprises information-theoretic methods that quantify neuronal selectivity, information content, redundancy, synergy, and directed information transfer in relation to sensory, behavioral, or network variables (Maffulli et al., 2022; Lorenz et al., 2025; Pospelov et al., 2026). The third characterizes the geometry and organization of population activity using dimensionality reduction, learned latent representations, neuronal manifolds, and activity-based sorting methods (Koh et al., 2023; Mitchell-Heggs et al., 2023; Schneider et al., 2023; Stringer and Pachitariu, 2024; Sun et al., 2024; Gerasimov et al., 2025a; Perich et al., 2025; Stringer et al., 2025; Raev et al., 2026). The fourth includes deep-learning and Transformer-based approaches, ranging from dataset-specific latent dynamical models to pretrained architectures and emerging foundation models intended to transfer learned representations across recordings, animals, tasks, or experimental conditions (Pandarinath et al., 2018; Ye and Pandarinath, 2021; Azabou et al., 2023; Ye et al., 2023; Antoniades et al., 2024). These categories are not entirely mutually exclusive, as learned latent-space methods may also use deep neural networks, but they differentiate the principal analytical outputs and provide the structure of the following discussion (Table 1).

**TABLE 1:** Summary of calcium imaging data analysis strategies and examples of their application to analysis of neuronal network dysfunction in Alzheimer’s disease (AD) mouse models.

Methodology	Computational approach	Main output	Analytical advantage	Demonstrated or potential relevance to AD mouse models studies
Signal quantification				
Mesmerize (Kolar et al., 2021)	Integrated processing, annotation, visualization, and interactive analysis	Extracted signals, annotated activity patterns, stimulus- or behavior-linked visualizations	Combines signal extraction with visual inspection and contextual interpretation of calcium imaging data	Can facilitate the analysis of calcium-imaging data from AD models by linking neuronal activity to experimental conditions and behavioral episodes
NeuroActivityToolkit (Gerasimov et al., 2023)	Quantitative analysis of processed miniscope calcium signals	Neuronal activations frequency, co-activation metrics, pairwise correlations	Converts processed calcium traces into comparable cellular and network activity metrics	Useful for detecting differences in excitability, coactivity, and network coordination between control and pathological conditions
Latent dynamics and functional networks				
Biophysical models and Bayesian inference (Rahmati et al., 2016)	Bayesian inference based on biophysical models of calcium dynamics	Reconstructed hidden neuronal dynamics from fluorescence traces	Accounts for calcium indicator kinetics, signal uncertainty, and the indirect nature of fluorescence recordings	May help distinguish genuine disease-related changes in neuronal firing from differences caused by indicator kinetics or measurement uncertainty
Inference of neuronal network spike dynamics and topology (Lütcke et al., 2013)	Spike dynamics inference and network topology reconstruction	Estimated spike-related dynamics and inferred functional topology	Moves from calcium-event description to reconstruction of hidden activity and network organization	May determine whether AD pathology affects functional-network topology in addition to overall activity levels
Information coding and neuronal selectivity				
NIT (Maffulli et al., 2022)	Information-theoretic analysis of neural population data	Entropy, mutual information, information carried by neural activity	Estimates what information neural activity contains about external or behavioral variables	May reveal whether AD pathology alters the information encoded about context, position, stimuli, or behavior
MINT (Lorenz et al., 2025)	Multivariate information-theoretic analysis	Multivariate coding metrics, information transmission measures, contribution of population interactions	Evaluates how information is distributed and transmitted across neural populations	May identify altered distributed coding, redundancy, synergy, or information flow in pathological circuits
INTENSE (Pospelov et al., 2026), arXiv	Information-theoretic detection of neuronal selectivity	Selective neurons, variable-specific encoding, mixed-selectivity profiles	Detects cells selectivity while accounting for temporal structure, delays, and correlations between behavioral variables	May reveal whether AD pathology changes neuronal selectivity to spatial context, movement, objects, or behavioral states
Population geometry, dimensionality reduction, and activity-based organization				
Dimensionality reduction of calcium-imaged neuronal population activity (Rubin et al., 2019; Koh et al., 2023; Mitchell-Heggs et al., 2023; Sun et al., 2024; Gerasimov et al., 2025a; Perich et al., 2025)	Dimensionality reduction and latent population analysis	Low-dimensional representations of population activity	Reveals hidden organization, trajectories, and separability of neural population states	Dimensionality-reduction approaches could be applied to calcium-imaging data in AD mouse models to identify disease-related changes in population organization; related manifold metrics may further quantify altered state geometry and trajectory stability
Rastermap (Stringer et al., 2025)	Activity-based ordering of neurons for structured raster visualization	Ordered raster maps, activity-defined neuronal groups, large-scale population patterns	Reveals population organization one-dimensional axis raster-like representation	May reveal disrupted coordination, abnormal synchrony, or altered sequential organization in AD models
CEBRA (Schneider et al., 2023)	Contrastive learning of joint neural-behavioral embeddings	Latent embeddings linking neural activity with behavior, position, or context	Builds an informative state space where neural and behavioral dynamics can be analyzed together	Could determine whether WT and AD-model animals occupy distinct neural–behavioral state spaces or show reduced stability of population representations
Deep-learning, transformer-based, and foundation-model approaches				
Foundation model of neuronal activity (Wang et al., 2025)	Foundation model trained on large-scale neuronal activity dataset	Predicted responses to new stimuli and anatomical conditions	Learns generalizable structure of neural population activity across datasets and conditions	May help to detect disease-related deviations in population response patterns from wild type mice
CalM (Xu et al., 2026), arXiv	Self-supervised foundation model based on a dual-axis Transformer, pretrained on large-scale calcium activity	Pretrained calcium-activity representations, population-dynamics forecasts, and behavioral-decoding outputs	Combines temporal representation learning, classification, and model-level attribution of informative activity features	May support transfer learning and cross-animal comparisons in relatively small AD mouse models calcium-imaging datasets
NEuRT/BERT-based transformer model (Raev et al., 2026)	BERT-based transformer architecture with self-attention mechanisms adapted for neuronal activity analysis	Reconstructed neuronal calcium traces, group classification, attention maps highlighting informative activity features	Combines latent representation learning with model interpretability and captures complex time-dependent interactions within neuronal populations	Applied to calcium-imaging recordings from AD mouse models to reconstruct neuronal activity, classify experimental groups, and identify activity features contributing to disease-model discrimination

The first group of methods addresses a fundamental limitation of calcium imaging: the fluorescence signal is an indirect and temporally smoothed reflection of neuronal electrical activity. An important analytical task is therefore not only to detect calcium events but also to reconstruct the hidden physiological processes underlying the observed fluorescence changes. In Rahmati et al. (2016), authors propose a Bayesian framework in which the measured fluorescence trace is considered the output of a biophysical model incorporating neuronal firing, calcium kinetics, and measurement noise. By fitting this model to the observed trace, the method estimates latent biophysical variables, including membrane potential and intracellular calcium dynamics, and reconstructs spiking or bursting patterns together with the uncertainty associated with these estimates. A related but distinct task was addressed by Lütcke et al. (2013), who used temporal relationships between calcium signals to construct functional networks. Neurons are represented as nodes and their estimated functional relationships as links. The resulting network can then be analyzed to determine how interactions are organized at the population level rather than considering cells as isolated sources of calcium events. Together, these approaches extend calcium imaging analysis from direct fluorescence quantification toward the inference of physiological and network processes underlying population activity.

Information-theoretic approaches address several related tasks, including the quantification of neuronal selectivity, the amount of information represented by individual neurons or populations, and the distribution and transfer of information within neuronal networks. The method described by Maffulli et al. (2022) estimates the amount of information that a neural signal carries about an external variable. Entropy is used to characterize variability in neuronal activity, while mutual information quantifies the association between activity and a selected sensory, spatial, or behavioral parameter. In practical terms, this method evaluates whether knowledge of a neuron’s activity or population reduces uncertainty about the variable of interest and is therefore primarily used to assess neural selectivity or information encoding. MINT is an independent toolbox for multivariate information analysis (Lorenz et al., 2025). In addition to measuring the information carried by individual cells or populations, it evaluates how this information is distributed across neurons, including redundant and synergistic population contributions. MINT can also be used to characterize directed information transfer between neurons or brain regions using measures such as transfer entropy. Its analytical scope includes both information encoding with respect to external variables and information flow within neuronal networks. As a further development of these information-theoretic principles, the open-source INTENSE framework was recently designed to detect neuronal selectivity to external and behavioral variables directly from continuous calcium fluorescence signals (Pospelov et al., 2026). It uses mutual information while accounting for temporal autocorrelation and delays associated with calcium indicator kinetics, applies cyclic-shift permutation tests to assess statistical significance, and employs conditional mutual information to distinguish genuine mixed selectivity from associations arising from correlations between behavioral variables. In its initial preprint report, INTENSE showed considerable potential for the analysis of neuronal selectivity under complex experimental conditions. Taken together, these methods allow neural activity to be considered not only as a set of events or pairwise correlations but also as a source of information about the environment, behavior, and the functional state of a neural network.

Another promising approach is the analysis of population activity structure using dimensionality reduction, neuronal manifold analysis (Mitchell-Heggs et al., 2023), and activity-based sorting (Stringer et al., 2025; Figure 2B). At each time point, the activity of a population containing N recorded neurons can be represented as a point in an N-dimensional space. Dimensionality-reduction methods transform these observations into a smaller number of coordinates while attempting to preserve selected properties of the original data, such as local neighborhoods, global distances, temporal continuity, or behaviorally relevant structure (Koh et al., 2023). The resulting representation can be used to quantify distances between population states, examine their trajectories, and evaluate the separation or overlap of states associated with different experimental conditions. Neuronal-manifold analysis is based on the observation that high-dimensional population activity often occupies a more restricted low-dimensional structure. Such manifolds may reflect coordinated neural dynamics, behavioral constraints, internal states, or combinations of these factors (Mitchell-Heggs et al., 2023). Their analysis can reveal changes in population geometry, dimensionality, stability, and trajectory structure that may not be apparent from cell-by-cell measurements. Koh et al. (2023) demonstrated that low-dimensional analysis can reveal structured population dynamics that are not apparent from cell-by-cell metrics. Accordingly, dimensionality reduction and neuronal manifold analysis are increasingly used to identify changes in the population coding under both normal and pathological conditions (Rubin et al., 2019; Koh et al., 2023; Mitchell-Heggs et al., 2023; Sun et al., 2024; Perich et al., 2025). As an example of such algorithms, CEBRA is a learned latent-space method that uses contrastive learning to construct neural embeddings aligned with time, behavior, or other experimental variables (Schneider et al., 2023). In contrast to conventional dimensionality-reduction methods that optimize a fixed geometric criterion, CEBRA learns a representation according to relationships defined by the training objective. It can therefore emphasize temporally or behaviorally relevant aspects of population activity, although the resulting embedding depends on the selected labels, sampling procedure, and model configuration. Rastermap (Stringer et al., 2025) addresses the same problem through a different representation. Rather than projecting population states into a new coordinate space, it reorders the rows of a raster plot so that neurons with similar activity patterns are placed next to one another. This makes coordinated groups of neurons, sequential activation patterns, and slow-population-wide modes more directly visible. Thus, Rastermap provides an interpretable visualization of population organization within the original activity raster, while dimensionality reduction methods offer quantitative low-dimensional representations of population states.

Artificial intelligence–based approaches can extend the analysis and representation of neuronal activity (Figure 2C), but their application remains constrained by a fundamental data bottleneck. Training expressive models requires large datasets, yet individual *in vivo* calcium imaging experiments yield comparatively few recordings. Moreover, data used to train a model cannot simultaneously serve as an independent test set, so the already-limited experimental data must be partitioned further, leaving less information for evaluation and biological analysis. Partly due to these limitations, early AI models for neuronal activity were typically optimized for an individual dataset or recording context and generalized poorly across different animals, brain regions, modalities, and experimental conditions. This limitation is well-illustrated by powerful but largely session-specific approaches: sequential autoencoders for latent population dynamics LFADS (Pandarinath et al., 2018), developed on intracortical electrode recordings and later adapted to two-photon calcium imaging as RADICaL (Zhu et al., 2022), the first neural-activity transformers (NDT) (Ye and Pandarinath, 2021), also developed on electrophysiology, contrastive learning of a neural encoder for latent embeddings (CEBRA) (Schneider et al., 2023). Autoencoder-based methods have also been used to detect anomalous miniscope calcium activity following acute stress (Gerasimov et al., 2024). Although such models can perform well within the datasets for which they are trained, they often require substantial adaptation when applied to new contexts.

Foundation models offer a potential solution to the limited transferability of dataset-specific models. These large-scale models are pre-trained on extensive, heterogeneous datasets and can subsequently be adapted to specific tasks with limited labeled data, yielding more flexible and transferable representations of complex biological signals. In the calcium-imaging domain specifically, this approach depends on the availability of large-scale annotated datasets — most notably MICrONS, currently the only dataset of its kind combining functional calcium imaging with structural connectivity at this scale: a functional connectomics dataset pairing two-photon calcium imaging of ∼75,000 excitatory neurons in awake mouse visual cortex with an electron-microscopy reconstruction of the same ∼1 mm^3^ volume (>200,000 cells, ∼523 million synapses). Such models have now been built directly on neuronal activity — in electrophysiology, POYO and the Neural Data Transformer 2 learn transferable representations of spiking activity across many sessions and animals (Azabou et al., 2023; Ye et al., 2023) — and, more relevant here, on calcium imaging itself: Neuroformer applies multimodal generative pre-training to two-photon visual-cortex calcium imaging analyzed jointly with visual stimuli and behavior (Antoniades et al., 2024); a foundation model of neural activity trained on the large-scale MICrONS two-photon dataset generalizes to new stimulus types and cortical locations (Ding et al., 2025; Wang et al., 2025); the recently proposed CalM model pre-trains directly on large-scale calcium traces across animals and sessions (Xu et al., 2026); and our own NEuRT model uses a transformer pre-trained on neuronal calcium activity (Raev et al., 2026). In the neuroscience field more broadly, the increasing availability of large-scale datasets such as MICrONS (Ding et al., 2025; Wang et al., 2025) provides the data foundation necessary to develop and apply such models. Nevertheless, disease- and hippocampus-specific resources of comparable scale remain scarce.

## 4 Traditional analytical approaches applications for *in vivo* calcium imaging data analysis for assessing neural circuits activity dysfunction in Alzheimer’s disease mouse models

In this section we discuss representative applications of traditional analysis approaches for characterizing neuronal network dysfunction in AD mouse models using two-photon imaging and miniscope recordings (Table 2). Alzheimer’s disease is a progressive neurodegenerative disorder and the leading cause of dementia, clinically defined by progressive memory impairment and cognitive changes (Dubois et al., 2021). Neuropathologically, AD is defined by the accumulation of amyloid-beta plaques (Hardy and Selkoe, 2002; Greenberg et al., 2020; Hampel et al., 2021; Nasb et al., 2024), intracellular neurofibrillary tangles formed of hyperphosphorylated tau (Muralidar et al., 2020; Rawat et al., 2022), and brain atrophy (Pini et al., 2016). These features are also accompanied by widespread synaptic degeneration (Popugaeva et al., 2015; Zhang et al., 2016; Subramanian et al., 2020; Meftah and Gan, 2023), neuronal loss (Goel et al., 2022) and reactive astrogliosis (Li et al., 2019; Gorina et al., 2022; Clayton and Liddelow, 2025). These pathological changes are associated with a wide range of downstream functional abnormalities, including calcium dysregulation (Bezprozvanny, 2009, 2026), altered synaptic transmission (Pelucchi et al., 2022) and progressive impairment of neuronal circuits function (Grieco et al., 2023).

**TABLE 2:** Summary of neuronal activity alterations identified by *in vivo* calcium imaging using event-based calcium activity analysis in Alzheimer’s disease mouse models.

References	Experimental conditions	Brain area and recorded cell’ types	Calcium imaging site	Activity alterations	Mechanism
Bai et al., 2017	Mouse line: APP/PS1 (3 momths); Method: two-photon calcium imaging under soluble Aβ oligomers toxicity; Conditions: head-fixed treadmill running	AAV-Syn-GCaMP6s expressed in layer 2/3 of cortex neurons	Soma/dendrite	Somatic hypoactivity; dendritic hyperactivity; prolonged Ca^2 +^ transients; reduced spine Ca^2 +^ and size	Soluble Aβ disrupts dendritic Ca^2 +^ signaling → synaptic depotentiation
Kuchibhotla et al., 2008	Mouse line: APP/PS1 (6–8 months); Method: two-photon calcium imaging, overall calcium levels or in 0–100 μm from Aβ plaques; Conditions: head-fixed anesthetized mice	AAV-YC3.6 expressed in layer five neurons of the neocortex	Soma/neurites	∼20% neurites with persistent Ca^2 +^ overload; spine loss; impaired synaptic integration	Plaque-associated chronic Ca^2 +^ elevation → structural degeneration
Lerdkrai et al., 2018	Mouse line: APP/PS1 (10–14 months); Method: two-photon calcium imaging, overall neuronal activity; Conditions: head-fixed anesthetized mice	OGB-1 labeling of the frontal/motor cortex neurons	Soma	↑ hyperactive neurons; ↑ Ca^2 +^ transient frequency	Intracellular Ca^2 +^ store dysfunction →↑ neurotransmitter release, NMDA activation
Lerdkrai et al., 2018	Mouse line: PS45 (6–14 months); Method: two-photon calcium imaging, overall neuronal activity Conditions: head-fixed anesthetized mice	AAV-hSyn-GCaMP6f expressed in layer 2/3 of the frontal/motor cortex neurons	Single neuron axon	Hyperactivity without plaques	↑ neurotransmitter release, NMDA activation
Korzhova et al., 2021	Mouse line: APP/PS1 (4–5 months); Method: two-photon calcium imaging, overall neuronal activity or in 0–80 μm from Aβ plaques; Conditions: awake head-fixed mice sitting in a restrainer	AAV-hSyn-mRuby2-GSG- GCaMP6s- expressed in layer 2/3 of the frontal cortex neurons	Soma	Gradual transition to hyperactive neurons (∼21%); stable elevated activity; ↑ synchrony	Progressive Aβ-driven activity imbalance
Busche et al., 2008	Mouse line: APP/PS1 (6–10 months); Method: two-photon calcium imaging, neuronal activity near Aβ plaques (0–140 μm); Conditions: anesthetized head-fixed mice by 0.8%–1.0% isoflurane	OGB-1 labelling of layer 2/3 cortical neurons	Soma	Hyperactive neurons clustering near plaques	Local Aβ microenvironment drives heterogeneous excitability
Algamal et al., 2022	Mouse line: APP/PS1 (8–11 months); Method: two-photon calcium imaging, overall neuronal activity or in 0–300 μm from Aβ plaques; Conditions: anesthetized head-fixed mice by 1.0% isoflurane	AAV-FLEX-jGCaMP7s expressed in layer 2/3 of the somatosensory cortex interneurons and AAV-CamKII-GCaMP6s expressed in layer 2/3 of the somatosensory cortex excitatory neurons	Soma	↓ excitatory neurons activity; ↑ SOM interneurons; PV dysfunction; ↓ functional connectivity	Interneurons subtype dysfunction → E/I imbalance
Busche et al., 2015	Mouse line: APP/PS1 (aged); Method: wide-field Ca^2 +^ imaging; Conditions: anesthetized head-fixed mice by 0.8%–1.0% isoflurane	OGB-1 labeling of anterior and posterior neocortical neurons	Total fluorescence signal from cortical areas	↑ activity fluctuations; ↓ cortex–hippocampus coherence	E/I imbalance → long-range disconnection
Hudry et al., 2019	Mouse line: APP/PS1 (8–10 and 18–20 months); Method: two-photon calcium imaging, overall neuronal activity regardless of Aβ plaques distance; Conditions: anesthetized head-fixed mice by 0.8%–1.0% isoflurane	AAV-CBA-YC3.6 expressed on primary visual cortex neurons	Soma	↑ “OFF” neurons; impaired stimulus-response coupling	Synaptic dysfunction driven by Aβ
Grienberger et al., 2012	Mouse line: APP/PS1 (8–10 months); Method: two-photon calcium imaging, visually evoked and spontaneous neuronal activity regardless of Aβ plaques distance; Conditions: head-fixed awake mice	OGB-1 labeling of layer 2/3 visual cortex neurons	Soma	Loss of tuning specificity; hyperactive neurons lose selectivity	Progressive network disorganization
Busche et al., 2019	Mouse line: APP/PS1-rTg4510 (6–12 months) and APP/PS1-rTg21221 (6–12 months); Method: two-photon calcium imaging, spontaneous neuronal activity under Aβ plaques pathology and tau load; Conditions: head-fixed awake mice	AAV-Syn-GCaMP6f expression in cortical layer 2/3 neurons	Soma	Widespread neuronal silencing; reduced Ca^2 +^ transients	Tau suppresses neuronal activity dominating amyloid-β effects
Zhang et al., 2023	Mouse line: 5xFAD (4–5, 8–10, and 14 months); Method: calcium imaging using miniscope, neuronal activity under Aβ plaques pathology; Conditions: freely behaving mice in open-field behavioral test	AAV-CaMKIIa-GCaMP6f expression in excitatory neurons of hippocampal CA1 area	Soma	↓ activity; unstable place fields; impaired spatial coding	Network instability → impaired ensemble coding
Lin et al., 2022	Mouse line: 3xTg-AD (3.6–6.5 and 18–21 months); Method: calcium imaging using miniscope, neuronal activity under Aβ plaques pathology and tau load; Conditions: freely behaving mice in two open-field arenas and linear track	AAV-CaMKIIa-GCaMP6f expression in excitatory neurons of hippocampal CA1 area	Soma	↑ activity and diffuse spatial tuning	Degraded population coding
Gerasimov et al., 2025b	Mouse line: 5xFAD (6.5 months); Method: calcium imaging using miniscope, neuronal activity regardless of Aβ plaques distance; Conditions: freely behaving mice in rounded arena and fear-conditioning behavioral testing	AAV-Syn-GCaMP6f expression in neurons of hippocampal CA1 area	Soma	↑ neuronal activity; ↑ bursts and hypersynchrony; altered functional connectivity	Hippocampal neuronal network hyperactivation and loss of connectivity profile
Erofeev et al., 2025	Mouse line: 5xFAD (5–6 months); Method: calcium imaging using miniscope, neuronal activity regardless of Aβ plaques distance; Conditions: freely behaving mice in fear-conditioning behavioral testing	AAV-Syn-GCaMP6f expression in neurons of hippocampal CA1 area	Soma	Impaired ensemble organization linked to behavior	Circuit disorganization

3xTg-AD, triple-transgenic Alzheimer’s disease mouse model; 5xFAD, five familial Alzheimer’s disease mutations mouse model; AAV, adeno-associated virus; Aβ, Amyloid beta; AD, Alzheimer’s disease; APP, amyloid precursor protein; APP/PS1, amyloid precursor protein/presenilin-1 mouse model; APP/PS1-rTg4510, double-transgenic mouse model combining APP/PS1 with tau overexpression; CA1, cornu ammonis area 1, subregion of the hippocampus; CaMKII, calcium/calmodulin-dependent protein kinase II; E/I, excitation/inhibition; GABA, gamma-aminobutyric acid; GCaMP, genetically encoded calcium indicator (various variants: 6f, 6s, 7s); OGB-1, oregon green BAPTA 488; PS45, presenilin-1 mutant mouse model; PV, parvalbumin; SOM, somatostatin; Syn, synapsin; YC3.6, yellow cameleon 3.6.

Experimental studies of Alzheimer’s disease rely on numerous transgenic and knock-in mouse models that have been developed to reproduce the key pathological and functional features of the disease. Given the wide variety of AD mouse models, which have been extensively reviewed elsewhere (Sasaguri et al., 2022; Yokoyama et al., 2022; Zhong et al., 2024), we provide only a brief overview of the models used in the studies discussed in this review. Presenilin (PSEN1 or PSEN2) mouse models express familial Alzheimer’s disease-associated mutations in presenilin genes, leading to altered γ-secretase activity (Bentahir et al., 2006) and disrupted intracellular calcium homeostasis (Tu et al., 2006). Presenilin-only models (for instance, PS45 mouse line) do not develop extracellular amyloid plaques, allowing investigation of amyloid-independent mechanisms of neuronal dysfunction, particularly alterations in calcium signaling and neuronal excitability (Elder et al., 2010). The APP/PS1 model (Jankowsky et al., 2004) expresses mutant human amyloid precursor protein (APP) and presenilin-1 (PS1), leading to progressive Aβ deposition beginning at 6 weeks of age in the cortex and 3–4 months in the hippocampal area (Radde et al., 2006). This model demonstrates early amyloid-driven pathology, including synaptic dysfunction (Gengler et al., 2010) and disrupted calcium homeostasis (Martinsson et al., 2022). The 5xFAD model (Oakley et al., 2006) carries five familial AD mutations in APP and PS1 genes, resulting in rapid Aβ accumulation, synaptic dysfunction, neuronal loss, and severe cognitive impairment (Forner et al., 2021). Its early and aggressive pathology makes it one of the most widely used model for investigating advanced amyloid-associated neuronal circuit dysfunction. In addition to impaired calcium signaling and beta amyloid plaques, another important feature of Alzheimer’s disease is deposition of hyperphosphorylated tau (Muralidar et al., 2020). Tau-bearing Alzheimer’s disease mouse models overexpress mutant human tau (for example rTg4510), producing progressive tau accumulation and neurofibrillary tangles by 4–5.5 months of age (Zhong et al., 2024). Unlike amyloid-based models, it primarily reproduces tau-mediated neurodegeneration and widespread neuronal hypoactivity. Although no single model fully captures the complexity of human AD, each demonstrates specific aspects of disease progression, making these models indispensable for *in vivo* calcium imaging studies of neuronal and network dysfunction.

The current review specifically focuses on alterations of neuronal circuit function revealed by *in vivo* calcium imaging. Neuronal activity alterations are complex and multi-scale phenomena that arise early in Alzheimer’s disease and evolve dynamically with pathology progression (Harris et al., 2020; Kazim et al., 2021; Targa Dias Anastacio et al., 2022; Xiong et al., 2023). A comprehensive body of evidence obtained via *in vivo* calcium imaging studies across different Alzheimer’s disease mouse models demonstrates alterations in both neuronal activity organization levels – from single neuron calcium activity dysregulation to total breakdown in neuronal circuitry functioning (Kuchibhotla et al., 2008; Busche et al., 2008, 2015, 2019; Grienberger et al., 2012; Bai et al., 2017; Lerdkrai et al., 2018; Hudry et al., 2019; Korzhova et al., 2021; Kazim et al., 2021; Algamal et al., 2022; Lin et al., 2022; Zhang et al., 2023; Erofeev et al., 2025; Gerasimov et al., 2025b). Using state-of-the-art techniques for recording neuronal calcium activity, these studies reveal complex, heterogeneous reorganization of neuronal activity patterns, where hyperactivity and hypoactivity at the single-cell level occur alongside profound impairments in neuronal ensemble coordination and connectivity.

### 4.1 Early stage neuronal dysfunction

At the earliest stages of pathology, even prior to plaques deposition, subtle but critical disturbances in neuronal activity emerge. In young APP/PS1 mice aged 3 months, *in vivo* two-photon calcium imaging of layer 2/3 cortical neurons expressing GCaMP6s revealed compartment-specific abnormalities of neuronal activity: somatic calcium activity was significantly reduced at rest, indicating hypoactivity, whereas dendrites exhibited abnormally prolonged and high-amplitude calcium transients during running (Bai et al., 2017). Critically, these altered dendritic calcium events were causally linked to soluble Aβ, as acute application of Aβ oligomers in wild-type mice reproduced the same prolonged calcium transients, providing direct experimental evidence for this mechanism. Importantly, affected dendritic branches displayed reduced spine calcium signals and decreased spine size, indicating early synaptic depotentiation and impaired plasticity, contributing to circuit destabilization at early stages in this transgenic mouse model. Moreover, in the same mouse line at a later stage (6–8 months), it was observed that approximately 20% of neurites exhibited persistent calcium overload, strongly dependent on their proximity to amyloid plaques. This chronic elevation of intracellular calcium disrupts the coordination between neurites and dendritic spines, thereby impairing synaptic integration. In parallel, the associated loss of spines directly links calcium dysregulation to structural degeneration at the single-neuron level (Kuchibhotla et al., 2008).

### 4.2 Progressive neuronal hyperactivity and activity heterogeneity

In APP/PS1 mice (10–14 months), *in vivo* two-photon calcium imaging revealed a significant increase in the fraction of hyperactive neurons, characterized by increased spontaneous Ca^2 +^ transient frequency, indicating a shift toward higher activity states. Similar alterations were observed in plaque-free PS45 mice (carrying the PS1_*G384A*_ mutations; 6–7 and 10–14 months of age), where presenilin mutation alone was sufficient to induce intrinsic neuronal hyperexcitability, evidenced by increased spontaneous Ca^2 +^ activity at the single-cell level. Mechanistically, these changes were linked to presynaptic dysfunction of intracellular Ca^2 +^ stores, as pharmacological depletion with CPA significantly reduced both the frequency and amplitude of Ca^2 +^ transients. At the circuit level, impaired cytoplasmic Ca^2 +^ enhanced neurotransmitter release and NMDA receptor activation, leading to increased network excitability. Notably, authors identified presynaptic calcium dysregulation as a major mediator of network dysfunction, as restoring Ca^2 +^ store function normalized both single-neuron and neuronal circuit activity (Lerdkrai et al., 2018). Longitudinal imaging of the same neuronal populations further showed that hyperactivity is a stable and a progressive hallmark of Alzheimer’s disease pathology in the cortex region. In APP/PS1 mice (4–5 months old), neurons gradually transitioned from intermediate to hyperactive states over weeks (Korzhova et al., 2021). At the single-neuron level, AD mice showed a significantly increased fraction of highly active neurons, defined by >4 Ca^2 +^ transients/min, and these aberrant activity levels were remarkably stable over time. Importantly, hyperactive neurons did not arise in a random manner, they gradually emerged from previously intermediately active neurons, with ∼21% transitioning to hyperactive states in APP/PS1 mice, demonstrating progressive activity dysregulation at the single-cell level. In parallel, at the circuit level, neurons showed increased pairwise correlations, indicating enhanced interneuronal functional connection and network-level dysfunction especially near amyloid plaques.

As pathology progresses, neuronal activity becomes increasingly heterogeneous. In APP/PS1 mice (6–10 months old), *in vivo* two-photon calcium imaging revealed a coexistence of hypoactive, normal, and hyperactive neurons within the same cortical region (Busche et al., 2008). Approximately 29% of neurons were functionally silent, whereas about 21% displayed abnormally high activity levels, while hyperactive neurons preferentially were found near amyloid plaques. This spatial clustering confirms that the β-amyloid microenvironment dramatically influences neuronal excitability.

### 4.3 Inhibitory circuit alterations

In parallel, inhibitory signaling alterations play a critical role in misbalancing neuronal network function. *In vivo* two-photon imaging of specific neuronal subtypes in APP/PS1 mice (8–11 months old) revealed that excitatory neurons in layer 2/3 of the somatosensory cortex (visualized using CAMKII-GCaMP6s) revealed an approximately 1.5-fold reduction in calcium events rate compared to WT littermates. This hypoactivity existed alongside hyperactivity of somatostatin-positive interneurons (visualized by SOM-jGCaMP7s), particularly near amyloid plaques, whereas parvalbumin-positive interneurons (visualized via PV-jGCaMP7s) showed reduced and altered activity (Algamal et al., 2022). This interneuron subtype-specific dysregulation results in a significant excitation/inhibition imbalance, leading to weakened functional connectivity, as evidenced by reduced pairwise correlations among excitatory neurons. Disruption of inhibitory neurons function also precedes amyloid plaques deposition.

Beyond local or region-restricted neuronal circuits disturbances, Alzheimer’s disease also affects large-scale brain network connectivity and functional coordination. Using large-scale calcium imaging, Busche et al. (2015) demonstrated pronounced connectivity abnormalities between long-range networks in APP/PS1 mouse. AD mice exhibited increased calcium transients frequency and altered dynamics, reflected by elevated activity fluctuations, indicating dysregulated excitability. At the circuit level, the most prominent deficit was a severe disruption of interregional regions coherence, particularly between cortex and hippocampus, indicating a breakdown of long-range functional connectivity. Topical application of gabazine (a selective, competitive antagonist of GABA_A receptors that blocks GABAergic synaptic transmission by binding to the GABA recognition site), rescued slow-wave activity and coordination between cortical regions, underscoring the pivotal role of E/I imbalance in both local and global circuit dysfunctions.

### 4.4 Degeneration of neuronal coding and neuronal networks functioning

At the sensory processing level, neuronal coding degenerates progressively. In APP/PS1 mice, two-photon imaging of the visual cortex revealed highly impaired neuronal responses to stimuli (Hudry et al., 2019). The authors observed a profound increase in “OFF-responding” neurons, indicating a disrupted coupling between sensory input and neuronal response. Similarly, longitudinal imaging in APP/PS1 mice demonstrated changes in the distribution of hypoactive, normal and hyperactive neurons in a disease-stage-dependent manner (Grienberger et al., 2012). Early-stage mice demonstrated normal neuronal orientation selectivity, while older animals exhibited a progressive loss of orientation selectivity, particularly in hyperactive neurons. At the same time, hypoactive neurons often became completely unresponsive to stimuli. At the circuit level, this alteration may translate into impaired population coding of visual information.

The interaction between Aβ-induced and tau pathologies further reorganizes neuronal activity patterns. While Aβ alone induces hyperactivity, tau pathology exerts an opposing effect. *In vivo* two-photon imaging in mouse models of Alzheimer’s disease expressing tau (rTg4510; 6–12 months old) or both tau and Aβ (APP/PS1-rTg4510 and APP/PS1-rTg21221; 6–12 months old) demonstrated that tau leads to profound neuronal silencing, with many neurons exhibiting no detectable calcium transients (Busche et al., 2019). Notably, when both pathologies are present, tau-driven hypoactivity dominates over Aβ-induced hyperexcitability, resulting in widespread suppression of cortex neurons activity. This effect is mediated by soluble tau species rather than neurofibrillary tangles, indicating that early tau pathology is sufficient to disrupt neuronal function.

At the level of regional neuronal circuits, particularly hippocampal ones, miniscope imaging studies in freely behaving mice provide further evidence of high-order violations in neuronal ensemble functioning. Longitudinal miniscope imaging of CA1 hippocampal neurons in 5xFAD mice revealed reduced activity rates and early hypoactivity, particularly during immobility, along with unstable and degenerate place fields (Zhang et al., 2023). These neurons exhibited reduced trial-to-trial consistency and impaired spatial tuning, leading to a deficit in population coding for object–location associations. Concurrently, in 3xTg-AD mice, hippocampal neurons displayed increased activity rates but reduced spatial specificity, with calcium events distributed diffusely rather than confined to well-defined place fields (Lin et al., 2022). Moreover, we recently demonstrated that at the circuit level, miniscope calcium imaging in freely behaving 6.5-months-old 5xFAD mice revealed pronounced hippocampal neuronal hyperactivity and disrupted functional connectivity of neuronal ensembles. These alterations were associated with impaired hippocampal neuronal circuitry functioning and cognitive deficits, supporting the idea that aberrant calcium-dependent network activity is a key feature of AD pathology in 5xFAD mice (Gerasimov et al., 2025b). Additionally, severe disruption of neuronal network function associated with cognitive performance was also observed in 5xFAD mice (Erofeev et al., 2025).

### 4.5 General patterns and sources of variability across calcium imaging studies of neuronal activity alteration in Alzheimer’s disease mouse models

Neuronal activity in calcium imaging studies was demonstrated to be profoundly disrupted in Alzheimer’s disease mouse models, although the direction and manifestation of these alterations depend strongly on disease stage, brain region, neuronal subtype, and pathological context. The strongest area of agreement is the progressive disruption of neuronal circuitry functioning. Most two-photon calcium imaging studies in the cortical areas in APP/PS1 mice consistently report the existence of abnormal neuronal activity patterns, including increased fraction of hyperactive neurons, enhanced synchrony, and impaired functional connectivity (Busche et al., 2008; Lerdkrai et al., 2018; Korzhova et al., 2021). Another point of consensus is that amyloid pathology promotes increasing heterogeneity of neuronal activity rather than uniform changes across all neurons. Multiple studies describe the coexistence of hypo-, normally and hyper-active neurons within the same cortical circuits, with hyperactive neurons preferentially located near amyloid plaques and progressively increasing in number during disease progression (Busche et al., 2008; Korzhova et al., 2021). Studies at early-stages of the Alzheimer’s disease in mouse model additionally demonstrate that soluble β-amyloid alone is sufficient to disrupt dendritic calcium transients before plaques deposition, indicating that neuronal dysfunction begins prior to overt plaque pathology (Bai et al., 2017). A further consensus concerns impaired neuronal information processing, not merely altered firing rates. Studies of the neuronal function in the visual cortex of AD mouse model demonstrate degradation of stimulus encoding accompanied by increased proportions of “OFF-responsive” neurons, loss of orientation selectivity, and impaired sensory coding (Grienberger et al., 2012; Hudry et al., 2019). In accordance, hippocampal miniscope studies demonstrate disrupted spatial representations, unstable place fields, and impaired neuronal ensemble organization during behavior (Lin et al., 2022; Zhang et al., 2023; Erofeev et al., 2025; Gerasimov et al., 2025b).

Despite these convergent findings, marked discrepancies remain regarding whether neuronal activity is predominantly increased or decreased. Numerous studies report cortical hyperactivity in APP/PS1 mice (Busche et al., 2008; Lerdkrai et al., 2018; Korzhova et al., 2021), whereas others observe somatic hypoactivity at early disease stages (Bai et al., 2017), reduced activity of excitatory neurons accompanied by interneuron dysfunction (Algamal et al., 2022), or widespread neuronal silencing in the presence of tau pathology (Busche et al., 2019). Similarly, hippocampal miniscope studies in 5xFAD mice report both reduced neuronal activity (Zhang et al., 2023) and pronounced hyperactivity (Gerasimov et al., 2025b). Several explanations may account for this heterogeneity. First, different mouse models represent distinct pathological mechanisms. APP/PS1 and 5xFAD models primarily examine amyloid pathology, whereas PS45 mice exhibit presenilin-dependent calcium dysregulation without plaques. In contrast, 3xTg-AD and APP/PS1-rTg4510 models additionally incorporate tau pathology, which promotes neuronal activity suppression (Busche et al., 2019). Second, disease stage appears to strongly influence neuronal activity. Early pathological stages are characterized by subtle compartment-specific abnormalities driven by soluble Aβ (Bai et al., 2017), intermediate stages often exhibit progressive neuronal hyperactivity and increasing synchrony (Lerdkrai et al., 2018; Korzhova et al., 2021), whereas advanced disease frequently shows greater activity heterogeneity, impaired neuronal coding, or hypoactivity associated with tau accumulation (Busche et al., 2019; Zhang et al., 2023). Third, imaging targets differ considerably among studies. Some investigations quantify somatic calcium activity, whereas others access dendritic, neuritic, axonal, or wide-field cortical fluorescence signals. Given the distinct calcium dynamics across these compartments, these measurements may capture different aspects of neuronal dysfunction. Fourth, methodological differences in imaging modality and behavioral conditions further contribute to variability. Most cortical studies employ two-photon imaging in head-fixed mice, often anesthetized mice, whereas hippocampal studies predominantly use miniscopes in freely behaving animals. Freely moving behavioral paradigms may capture experience-dependent neuronal ensembles changes and spatial coding, while anesthesia suppresses brain activity and may alter network dynamics (Wang et al., 2025), making direct comparisons between studies challenging.

Finally, methodological variability: across calcium sensors (OGB-1, YC3.6, GCaMP6s/f, jGCaMP7s), cell types (non-selective neuronal recordings, in excitatory neurons, somatostatin positive and parvalbumin positive interneurons), and analytical metrics (frequency, hyperactivity, synchrony, connectivity, tuning, place fields, ensemble organization), may contribute to discrepant findings. Thus, studies often measure different features of dysfunction rather than a shared endpoint. Nonetheless, a convergent picture emerges: AD progressively disrupts calcium-dependent circuit function. Although the polarity of activity changes remains debated, there is consensus that neuronal activity becomes increasingly heterogeneous, network coordination degrades, and coding fidelity declines.

## 5 Machine learning and artificial intelligence applications for calcium imaging analysis in Alzheimer’s disease mouse models

As outlined in the previous chapter, neuronal activity is profoundly disrupted across multiple brain regions in Alzheimer’s disease mouse models. Here, we discuss the application of AI-based analytical methods to characterize hippocampal network dysfunction in AD. Using *in vivo* miniscope calcium imaging and conventional calcium event analysis, our group previously demonstrated that 5xFAD mice exhibit hippocampal hyperactivity, aberrant neuronal co-activation, reduced circuit stability, impaired functional connectivity, and altered recruitment of neuronal ensembles during memory-related behaviors such as fear conditioning (Gerasimov et al., 2025b). Building on the same dataset, we further combined deep-learning-based behavioral tracking with neuronal manifold analysis to investigate hippocampal population dynamics (Gerasimov et al., 2025a). AI-driven pose estimation and behavioral classification were integrated with calcium imaging data to construct low-dimensional manifolds representing neuronal activity across behavioral states (Figure 2B). Unlike conventional approaches focused on individual neurons or pairwise correlations, manifold learning captures coordinated population dynamics and reveals the organization, temporal evolution, and stability of neural activity patterns (Mitchell-Heggs et al., 2023). This systems-level perspective is particularly valuable in AD, where cognitive impairment arises from disrupted ensemble coordination rather than single-neuron dysfunction alone. In particular, using dimensionality reduction approach, hippocampal activity in 5xFAD mice was clearly separated from that of wild-type animals and exhibited reduced intracluster distances, possibly reflecting lower variability of neuronal network states and altered network organization. Behavioral state-specific analyses further revealed markedly smaller manifold areas (the low dimensional area occupied by all embedded points in the t-SNE space) during running, sitting, and grooming. This reduced embedding-space spread may be consistent with lower diversity of neuronal population states, in line with the pattern of neuronal hyperactivity described above (Figure 2B). These observations are consistent with our previous findings (Gerasimov et al., 2025b) and with reports of neuronal hyperexcitability and disrupted neuronal coding in AD models (Lin et al., 2022). Finally, encoder-based classification of manifold-derived features successfully distinguished untreated 5xFAD mice from WT controls but failed to separate NDC-9009-treated [a positive allosteric modulator of SERCA pump, that was shown to reduce synaptic degeneration, behavioral impairments and β-amyloid load in the Alzheimer’s disease mouse models through stabilization of cytosolic Ca^2+^ levels (Dahl et al., 2023; Rakovskaya et al., 2023; Dahl and Bezprozvanny, 2024)] 5xFAD mice from WT littermates. These findings might suggest that NDC-9009 treatment shifted the distribution of hippocampal circuit activity patterns toward a WT-like configuration, though manifold-based representations cannot be considered as direct evidence of biological restoration.

Further, in the Raev et al. (2026), a transformer architecture for neuronal activity interpretation was established (Figure 2C). Transformers, originally developed for natural language processing, are particularly well-suited for modeling long-range temporal dependencies in sequential data and have found wide application in neuroscience, neurology, and psychiatry fields (Cong et al., 2024; Nerella et al., 2024). In calcium imaging datasets, neuronal activity exhibits complex and highly dynamic temporal patterns distributed across large neuronal populations, making transformer-based frameworks especially appropriate for this type of analysis. NEuRT was pre-trained on the large-scale MICrONS dataset (Ding et al., 2025; Wang et al., 2025) and demonstrated strong generalization performance by precisely reconstructing calcium signal traces across both visual cortex two-photon imaging (Ding et al., 2025; Wang et al., 2025) and hippocampal miniscope datasets from our prior study (Gerasimov et al., 2025b). Conceptually, this was the first demonstration that transformer architectures can be applied to calcium-imaging analysis at scale — enabled both by the availability of a large two-photon dataset for pre-training and by the confirmation that the learned representations transfer from two-photon to miniscope recordings, bridging two imaging modalities (Figure 2C). The model was subsequently fine-tuned to classify wild-type and 5xFAD Alzheimer’s disease model mice based on hippocampal neuronal activity, revealing robust disease-related differences in network dynamics. To identify which signal features drove the classification, the input components — the mean and standard deviation of the activity of the near- and far-located neurons relative to each analyzed neuron — were ablated (set to zero) group by group, and the resulting change in classification accuracy was used to pinpoint the most informative components. Attention rollout is a *post hoc* attribution method that combines attention matrices across transformer layers to estimate the cumulative contribution of each original input token to a selected output. It produces a relevance vector or map that can be summarized across neurons or time, but these values reflect model-level attribution rather than causal neuronal influence or functional connectivity. Taken together, component ablation and attention rollout expose the model’s decision process and identify the activity features that distinguish wild-type from 5xFAD neuronal networks, which could serve as an interpretable characterization of the underlying pathological alterations. In particular, it was demonstrated that the key features underlying neuronal activity alterations in 5xFAD neuronal network were associated not with the spatial organization of activity between closely or distantly located neurons, but rather with changes in overall mean activity levels and the magnitude of activity fluctuations (Figure 2C). These findings are consistent with the neuronal hyperactivity and aberrant neuronal co-activation patterns reported in Alzheimer’s disease mouse models, rather than with alterations confined to individual neurons (Figure 2C). These observations may strengthen the idea that global shifts in excitability and variability may play a more critical role in network dysfunction than spatial coordination patterns within the hippocampal circuitry. Together with the observation that shuffling the temporal order of these values disrupted classification performance, these ablation findings indicate that the model relies on genuine temporal dynamics among mean-activity values, rather than on a single static summary statistic computed over the whole session. Its access to spatial information, however, is constrained by the input representation itself: activity is aggregated into only two spatial groups near- and far-located neurons rather than resolved at the level of individual neuron pairs, so the model’s apparent spatial sensitivity should be understood as reflecting this coarse, population-level grouping rather than fine-grained neuronal connectivity. Together, these results represent an early demonstration that transformer-based architectures can extract temporally structured, disease-relevant signatures from population-level calcium activity, motivating further exploration of such models and of alternative architectures capable of resolving individual-neuron interactions as calcium-imaging datasets grow in scale.

Another important future direction is to understand brain regions function and animal behavior. This could be achieved by incorporating behavioral data as an additional dimension into large artificial intelligence models, which could be crucial for understanding the pathological shifts in the neuronal activity linked to behavior. In Gerasimov et al. (2025a), we showed that manifolds constrained on hippocampal neuronal network activity in Alzheimer’s disease mouse model exhibit altered composition across behavioral epochs. However, these findings represent only an initial step toward understanding the interplay between circuit and behavioral dysfunction in AD mouse model, and should be extended to other behavioral tests and brain regions. A particularly promising direction is the use of explainable multimodal transformer architectures that jointly process neuronal activity and synchronized behavioral variables, such as locomotion, spatial position, task performance, and exploratory behavior. Through attention-based mechanisms, such models may identify neurons, neuronal ensembles, and time periods most strongly associated with specific behavioral states or cognitive processes. This approach could reveal disease-associated alterations in neuronal–behavioral coupling and provide sensitive biomarkers for evaluating therapeutic interventions.

Despite these advantages, manifold-learning and Transformer-based analyses carry important limitations that should be weighed against the classical approaches discussed in the previous chapter.

Low-dimensional representations are not method-independent: their geometry varies with data preprocessing, the definition of temporal windows, the choice of dimensionality-reduction algorithm linear (e.g., PCA, ICA) versus non-linear (e.g., t-SNE, UMAP) and its hyperparameters, so the same calcium-imaging session can yield substantially different embeddings. Consequently, distances, cluster compactness, or occupied areas measured in t-SNE or UMAP space should not be interpreted as intrinsic geometric properties of the original high-dimensional neuronal activity space. Because manifold construction typically relies on comparatively small, single-laboratory sessions, limited and heterogeneous datasets may additionally produce unstable embeddings, increase susceptibility to overfitting, and reduce the generalizability of downstream classifiers; unlike deep-learning pipelines, however, most manifold-learning methods are computationally lightweight and do not require specialized hardware. Reproducibility therefore requires validation across animals, recording sessions, imaging systems, calcium indicators, behavioral paradigms, and, where possible, independent laboratories, together with consistent reporting of preprocessing and embedding parameters. Nevertheless, when such parameters are applied consistently throughout a study, manifold-based approaches remain a powerful means of uncovering the organization and dynamics of neuronal circuits under both physiological and pathological contexts (Levy et al., 2023; Esparza et al., 2025).

Transformer-based models introduce additional challenges related to the amount and representativeness of training data and to computational requirements: large-scale pretraining may require substantial data-storage and GPU resources, potentially limiting the accessibility of these approaches to smaller laboratories and complicating independent replication. In the case of NEuRT, the reported limitations include sensitivity to the number of neurons recorded in a session, possible loss of neuron-level information resulting from aggregation of population activity, dependence on model hyperparameters and class balance, and potential shortcut learning associated with strongly separable disease-related features. A further, more general limitation concerns interpretability: attention maps and attention rollout provide model-level attribution by indicating which input components or temporal intervals influenced a prediction, but they do not by themselves establish causal neuronal interactions, functional connectivity, or biological mechanisms.

Accordingly, results obtained using manifold- and Transformer-based approaches should be interpreted together with classical calcium-imaging metrics and supported by held-out-animal testing, external validation, ablation analyses, and transparent reporting of preprocessing and model settings.

## 6 Future perspectives of ML- and AI-driven calcium imaging analysis and its implications for studies of neuronal network function and dysfunction

The integration of *in vivo* calcium imaging into Alzheimer’s disease research has substantially advanced our understanding of the transition from molecular pathology to neuronal and circuit dysfunction. Despite the remarkable progress in this field, several important conceptual and methodological limitations remain unresolved.

A central methodological question in *in vivo* calcium imaging is whether calcium signals can be considered reliable proxies for neuronal firing, particularly in the context of AD. While genetically encoded calcium indicators enable monitoring of activity in large-scale neuronal populations, Ca^2 +^ signals represent an indirect measure of neuronal excitability and are subject to several important limitations. First, the temporal resolution of calcium imaging is inherently lower that of electrophysiological recordings, owing to the kinetics of calcium influx, buffering, and indicator response (Wei et al., 2020; Siegle et al., 2021). Rapid neuronal spiking may therefore be underestimated or temporally blurred. Second, indicator saturation can occur during high-frequency activity episodes, compressing differences between firing rates and potentially masking pathological hyperexcitability. Third, the relationship between Ca^2 +^ signal amplitude and spike number is non-linear (Deneux et al., 2016), particularly in diseased tissue where calcium homeostasis is altered. This non-linearity complicates quantitative comparisons across conditions and models. These limitations are especially critical in Alzheimer’s disease, where abnormal calcium homeostasis and disrupted intracellular signaling may further distort the relationship between fluorescence dynamics and actual neuronal activity.

Additional technical factors also constrain the interpretation of *in vivo* calcium imaging data. Neuropil contamination, arising from out-of-focus fluorescence or signals from nearby processes, can artificially affect somatic activity measurements. Furthermore, calcium signals differ substantially between cellular compartments: somatic signals primarily reflect action potential-associated calcium influx, whereas dendritic signals may encode synaptic input or local processing (Ali and Kwan, 2019). In AD, where dendritic pathology is prominent, these compartment-specific differences become particularly relevant and may contribute to divergent interpretations across studies. Importantly, disease-related alterations in calcium handling, including changes in channels expression, intracellular buffering, and organelle function, may further confound the relationship between Ca^2 +^ signals and underlying electrical activity (Bezprozvanny, 2009, 2022, 2026; Cascella and Cecchi, 2021).

From a computational perspective, classical analytical methods remain essential for robust calcium signal preprocessing, including motion correction (Pnevmatikakis and Giovannucci, 2017), ROI extraction (Lu et al., 2018; Zhou et al., 2018; Dong et al., 2022), spike inference (Pachitariu et al., 2018), and validation of biological findings. Modern ML and AI approaches, in their current form, carry important limitations that restrict their widespread application in neuroscience research (Abrams et al., 2026). One principal challenge is the relatively small size and high heterogeneity of neuroscience datasets. Machine learning models trained on limited experimental data may experience overfitting and poor generalization across laboratories due to differences in imaging systems, calcium indicators, behavioral paradigms, and animal models. A further important shortcoming is interpretability: although autoencoders and transformers demonstrate strong performance in identifying hidden patterns within neuronal activity data (Nerella et al., 2024), the biological relevance of latent representations frequently remains ambiguous. In many instances, these representations may capture dependencies without direct correspondence to biologically meaningful neuronal states. Consequently, they require careful expert evaluation and comparison with well-established classical methods during interpretation. This consideration is particularly pertinent in Alzheimer’s disease research, where distinguishing primary pathological mechanisms from compensatory responses remains challenging. Explainable AI methodologies, including attention-based models (Raev et al., 2026), interpretable latent-space analysis, and, as discussed in more detail below, graph-based architectures that make circuit structure explicit, may partially address these limitations; their application to *in vivo* calcium imaging, however, remains in its early stages.

An additional challenge is the handling of multimodal neuroscience datasets, which often include not only calcium recordings but also animal behavioral and time steps of various experimental events. For example, the MICrONS dataset provides unprecedented opportunities for integrating neuronal activity with synaptic connectivity and structural organization (Ding et al., 2025; Wang et al., 2025). Future advances in multimodal foundation models may help establish mechanistic relationships between neuronal structure, connectivity, activity dynamics, and behavioral outcomes under both physiological and pathological conditions.

Nevertheless, the studies discussed above illustrate that modern, complex analysis of high-dimensional *in vivo* calcium imaging data should be based not only in classical signal-processing approaches, which remain essential for quantifying neuronal activity parameters, but also lean on data-driven machine learning or artificial intelligence frameworks. As demonstrated previously, autoencoder-based models enable sensitive anomaly detection and latent feature extraction, manifold learning captures population-level organization of neuronal circuits, and transformer-based architectures provide explainable modeling of complex temporal neuronal dynamics. Importantly, these approaches are not intended to replace classical calcium imaging data analytical methods, which remain the “gold standard” for interpreting neuronal activity data in any form, but rather to complement and extend them. Traditional methods hold a vital role in signal preprocessing, motion correction, ROI extraction, and validation of biological findings. Concurrently, ML- and AI-driven approaches may offer advantages for analyzing large-scale, high-dimensional datasets, detecting subtle pathological signatures, and uncovering hidden principles of circuit organization. In Alzheimer’s disease research in particular, these methods could provide powerful tools for identifying early network dysfunction, characterizing circuit instability, and linking neuronal population dynamics to behavioral impairments at increased resolution.

**FIGURE 3:**
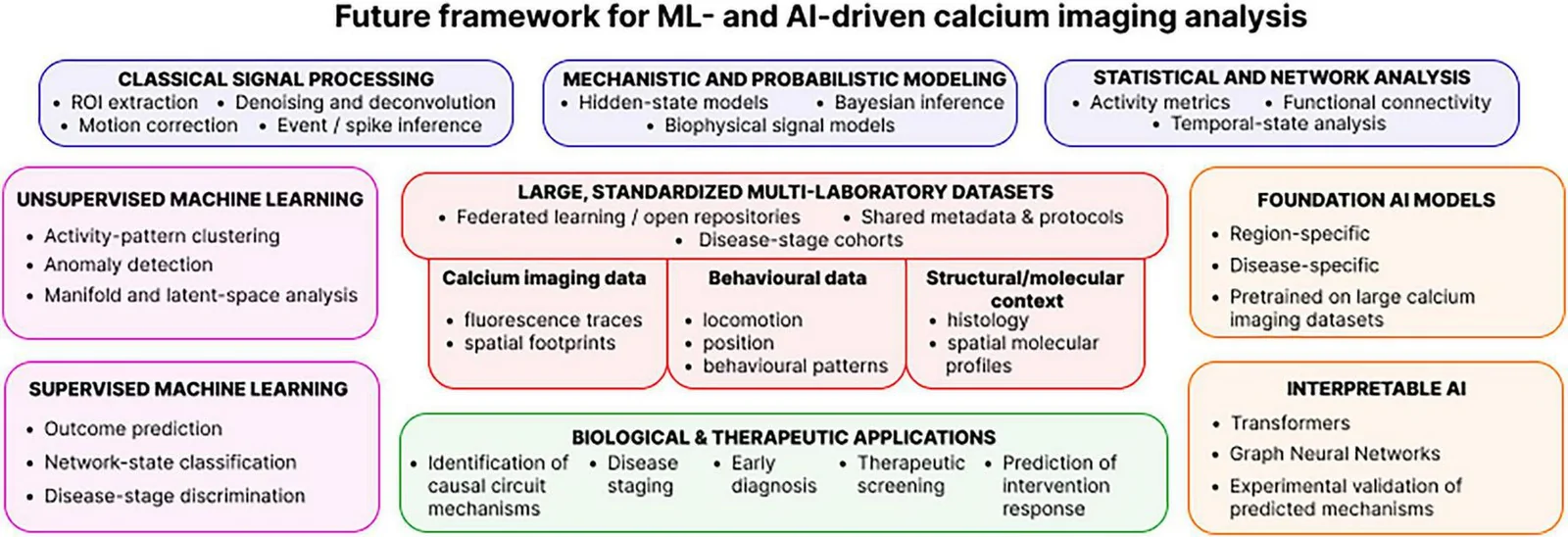
Future framework for machine learning-based calcium imaging analysis in mouse models of neurodegenerative disorders. Calcium imaging and behavioral data are first processed using classical signal-processing, statistical, network, and mechanistic modelling approaches. Supervised and unsupervised machine-learning methods subsequently enable network-state classification, disease-stage discrimination, neuronal ensemble discovery, latent-space analysis, and anomaly detection. The development of large, standardized multi-laboratory datasets will support region- and disease-specific foundation models, whereas interpretable AI approaches may identify the neurons, connections, activity patterns, and time periods most relevant to model predictions. Integration of neuronal activity with behavioral, structural, molecular, and histopathological information may provide a unified representation of circuit dysfunction. These computational advances could facilitate early disease staging, identification of mechanistic biomarkers, therapeutic screening, prediction of treatment responses, and translation of mouse calcium-imaging signatures to human EEG- and fMRI-based biomarkers.

Here we summarize possible future directions in calcium imaging data analysis in the context of AD study (Figure 3).

### 6.1 From description to disease staging and therapeutic readout

To date, AI-based analyses of calcium imaging in AD have been used largely descriptively to ask whether wild-type and AD networks differ. Their greater, and still under-exploited, value is interventional: network-level signatures can serve as quantitative, sensitive biomarkers for staging disease and, in particular, for screening therapeutics, offering a complementary, higher-resolution readout alongside behavioral, PET, and fluid biomarkers of disease pathology. Resolving disease stages, however, is methodologically constrained: stable longitudinal imaging of the same neurons through a GRIN lens can typically be sustained for only weeks, and rarely beyond a month, so tracking a full disease trajectory within a single animal is seldom feasible. A more realistic strategy is to assemble cross-sectional cohorts spanning defined disease stages and, most valuably, to include the prodromal window, before amyloid plaques and overt structural changes become detectable; this is both the most informative regime for understanding disease initiation and precisely the one in which highly sensitive, data-driven methods may be better positioned to resolve the subtle network alterations that classical type of analyses may miss. The reasoned next step is to extend this framework from characterizing disease to evaluating treatment via the same network signatures to test whether, and at which stage, a therapeutic intervention restores circuit function, ideally identifying the latest window in which intervention is still effective.

### 6.2 The data bottleneck: building toward region- and disease-specific models

The rate-limiting step for AI and ML based methods application is data amount, rather than models’ architecture. Foundation models succeed in vision and language applications because of web-scale corpora; systems neuroscience has nothing comparable, and even MICrONS, transformative as it is, samples healthy visual cortex neurons dynamics rather than hippocampal or disease-associated ones. Near-term progress therefore depends less on novel architectures than on assembling large, standardized, openly shared, multi-laboratory calcium-imaging datasets. They should be accompanied with harmonized metadata and the data-sharing norms the field still largely lacks; where data ownership or privacy impede pooling, while federated learning offers a route to training shared models without centralizing raw recordings. For Alzheimer’s disease specifically, such datasets should be also aligned (Klumpp et al., 2025) with memory-specific behavioral recordings, so that neuronal population activity can be tied to the encoding and retrieval processes the disease disrupts (Puzzo et al., 2014; Ghafarimoghadam et al., 2022). As region- and disease-specific data of this kind will be accumulated, it should become feasible to pre-train foundation models directly on hippocampal and AD-state neuronal calcium activity. This approach would help overcome the substantial, yet rarely quantified, domain shift that occurs when models pretrained on wild-type cortical neuronal activity are applied to hippocampal or disease-state data. Consequently, claims of model generalization across brain regions and disease states should be interpreted with caution unless they are explicitly validated through precise benchmarking. At present, however, such datasets remain scarce, and building them should be treated as a deliberate and prioritized goal rather than something expected to emerge naturally over time.

### 6.3 Interpretability and multimodal integration as the design criteria

The next generation of models should be guided by two properties rather than by predictive accuracy alone. The first is interpretability in a strong, causal sense. The second is the ability to combine several types of data within a single model.

Interpretability of this kind must go beyond correlation, and it can be built into a model in two complementary ways. The first is to choose architectures which internal computations are inspectable: transformers expose attention weights that show which neurons and time points the model uses for a given prediction, while graph neural networks make the circuit explicit, since their nodes are neurons and their edges are functional or anatomical connections. The second is to apply *post hoc* interpretation tools to the trained model, for example attention and saliency maps that rank the most influential neurons or activity patterns, feature attribution methods that quantify each input’s contribution, and structured analysis of the latent space (such as linear probes or low-dimensional projections) that links learned representations to known biological variables. These methods indicate what the model relies on, but not which neural events actually cause an outcome. Establishing causality therefore requires experimental validation, ideally by perturbing the model-identified neurons or patterns (optogenetically, chemogenetically, or pharmacologically) and testing whether circuit function changes as predicted. Until this step is taken, attention weights and similar signals should not be read as mechanism on their own.

The second criterion, multimodal integration, is served by the same architectures but draws on different strengths. Transformers can fuse heterogeneous inputs by representing them as a common sequence of tokens, which makes them well-suited to modeling neural activity together with behavior and additional data streams. Explainable transformer architectures can be integrated with behavioral data by jointly processing neuronal activity recordings and synchronized behavioral variables, such as locomotion, spatial position, task performance or exploratory behavior. In this concept, attention mechanisms enable the identification of specific neurons, neuronal ensembles, and time periods that contribute most strongly to particular behavioral states or cognitive processes. As noted above, graph neural networks are complementary: beyond encoding connectivity in their edges, they can carry molecular or morphological features on each node, so that structural and functional information enter the same model. The aim is not to declare one architecture universally superior, but to select models on these two axes, interpretability and multimodal integration, so that structure, activity, and behavior jointly shape a single shared representation instead of being analyzed in parallel.

### 6.4 True multimodal integration: structure and neuronal network and behavior

Building on the multimodal-integration criterion introduced above, the richest opportunity is to integrate calcium activity with synaptic connectivity (e.g., MICrONS), spatially resolved molecular state (spatial transcriptomics), and synchronized behavior within a single model – precisely the integrative task the architectures described above are suited. Particularly, in AD such models could link the amyloid-plaque microenvironment to single-cell excitability and, in turn, to emergent network states, and could ultimately support *in silico* experiments that predict circuit responses to perturbation before they are performed. Anchoring these models to behavioral and cognitive outcomes is essential: the key challenge is to determine at which stage of information processing and circuit state dysfunction emerges, requiring experimental paradigms that enable the integration of neuronal activity measurements with cognitive behavioral paradigms. Water-based tasks such as the Morris water maze (Redish and Touretzky, 1998; Vorhees and Williams, 2006) are poorly suited to simultaneous miniscope recording; dry, arm-based paradigms like the Y-maze (Kraeuter et al., 2019) and fear conditioning (Curzon et al., 2009) are far more tractable for aligning calcium dynamics with discrete memory events. A second, currently under-used dimension is *post hoc* histology. Correlating the same *in vivo*-imaged neurons to immunohistochemistry for amyloid plaques, and to dendritic and spine morphology after imaging, would allow local plaque burden and structural state to be linked to functional signatures on a per-cell, per-animal basis. The principal obstacle is technical: handling these samples and matching individual imaged cells to their histological counterparts remains demanding within a standard laboratory workflow and is itself a registration-and-matching problem to which computational methods could contribute.

### 6.5 The translational gap is the hardest frontier

All of the above remains in mouse models. The honest, unresolved question is whether these network signatures have human analogues accessible through non-invasive measures like cortical hyperexcitability, network desynchronization, and altered functional connectivity reported with EEG (Tsolaki et al., 2014; Chetty et al., 2024) and fMRI (Sperling, 2011; Alorf and Khan, 2022; Alarjani and Almarri, 2025). Aligning mouse-derived circuit signatures with human biomarkers, and testing whether AI models trained in one species inform the other, is where the clinical value of these studies will be decided.
